# Position- and quantity-dependent responses in zebrafish turning behavior

**DOI:** 10.1038/srep27888

**Published:** 2016-06-13

**Authors:** Keiko Umeda, Toru Ishizuka, Hiromu Yawo, Wataru Shoji

**Affiliations:** 1 Frontier Research Institute for Interdisciplinary Sciences, Tohoku University, Sendai, 9808578, Japan; 2 Department of Developmental Biology & Neuroscience, Tohoku University Graduate School of Life Sciences, Sendai, 9808577, Japan; 3 Department of Project Programs, Institute of Development, Aging and Cancer, Tohoku University, Sendai, 9808575, Japan

## Abstract

Neural reflexes are stereotypical automatic responses often modulated by both intrinsic and environmental factors. We report herein that zebrafish larval C-shaped turning is modulated by the stimulated position of Rohon-Beard (RB) neurons. Targeted stimulation of more anterior RB neurons produces larger trunk flexion, which anticipates adult escape behavior by coordinated turning toward the appropriate direction. We also demonstrated that turning laterality varies with the numbers of stimulated neurons. Multi-cell stimulation of RB neurons elicits contralateral turning, as seen in the touch response to physical contact, while minimum input from single-cell stimulation induces ipsilateral turning, a phenomenon not previously reported. This ipsilateral response, but not the contralateral one, is impaired by transecting the ascending neural tract known as the dorsolateral fascicule (DLF), indicating that two, distinct neural circuits trigger these two responses. Our results suggest that RB neurons transmit the position and quantity of sensory information, which are then processed separately to modulate behavioral strength and to select turning laterality.

Sensory-motor coordination adapts animal motilities to their perceiving environment. When aquatic vertebrates sense water vibration from an oncoming threat, they turn to the contralateral side by adopting a C-shaped body curvature of variable strength to elicit adequate escape trajectories. For example, sensory stimulus from the anterior evokes a large turning curvature to move toward the side opposite the threat, while stimulus from the posterior elicits small changes in direction to swim forward^1^,^2^. It is thought that during these behaviors, sensory information is integrated in brainstem reticulospinal (RS) networks including Mauthner neurons, to activate contralateral motor neurons to the appropriate extent. Although RS neurons and their circuits are well conserved in lower vertebrates^3^,^4^,^5^, it remains unclear how spatial perception is transformed to activate such motor coordination.

Zebrafish larvae develop rapidly and acquire stereotyped motility patterns, which provide model networks for studying neural circuits developing from their conserved simple backbones. The first embryonic reflex is the touch response and is observed at 21 hpf (hours post-fertilization), whereby contact on the epidermis elicits body twists due to trunk muscle contraction^6^. This response becomes much faster at 27 hpf, and produces contralateral body flexion that is often associated with alternating tail flips in a process resembling the adult escape behavior composed of C-Shaped turning and brief swimming^7^,^8^. In this larval response, tactile stimuli on the trunk are detected by Rohon-Beard (RB) mechanosensory neurons, whose peripheral arbors cover the entire trunk epidermis by overlapping with neighboring cell arbors. RB cell bodies form a pair of longitudinal columns within the dorsal spinal cord and their anterior-posterior (A-P) sequence comprising the receptive field potentially conveys positional information, as observed in other somatosensory systems^9^; however, little is known about how stimuli on RB neurons are treated differently depending on their A-P position. Previous anatomical and electrophysiological investigations suggest that RB sensory information is transmitted beyond the spinal cord via either side of the ascending sensory tract called the dorsolateral fasciculus (DLF). RB central axons fasciculate on the ipsilateral side to form the DLF, and their ascending branches extend up to the hindbrain^10^,^11^. In addition, RB central axons form synaptic contacts with CoPA (commissure primary ascendance) neurons, from which the post-commissural axons merge with DLF on the contralateral side, enabling extension beyond the spinal cord^10^,^12^,^13^. In the hindbrain, RS neurons are activated in response to tactile stimuli on the trunk, implicating these neurons in production of the C-shaped turns^14^,^15^. On the other hand, transection studies at the hindbrain level suggested that supra-spinal components were less involved in larval escape behavior, and that rostral spinal cord instead constitutes sufficient circuits for the response^8^,^16^. Thus, neural circuits eliciting and regulating a larval turning response appear to be redundant, and it remains unclear how these redundant circuits distribute their roles under physiological conditions.

In this study, we show that the turning response of zebrafish larvae is regulated according to their sensory receptive position. Specifically, RB neurons innervating a more-anterior region produce more extensive C-shaped body turning, suggesting the A-P position is perceived to control behavioral strength. We also demonstrate that stimulation of multiple RB neurons, rather than a single neuron, produce contralateral turning via intraspinal circuits. Nonetheless, stimulation of a single RB neuron elicits an ipsilateral turning response, which requires supraspinal circuits. Thus, our results suggest that sensory information from RB neurons is transmitted to supraspinal networks, while the robust escape response is triggered by intraspinal circuits that require multi-cell input from RB neurons.

## Results

### Behaviors elicited by single RB neurons

**Figure 1:**
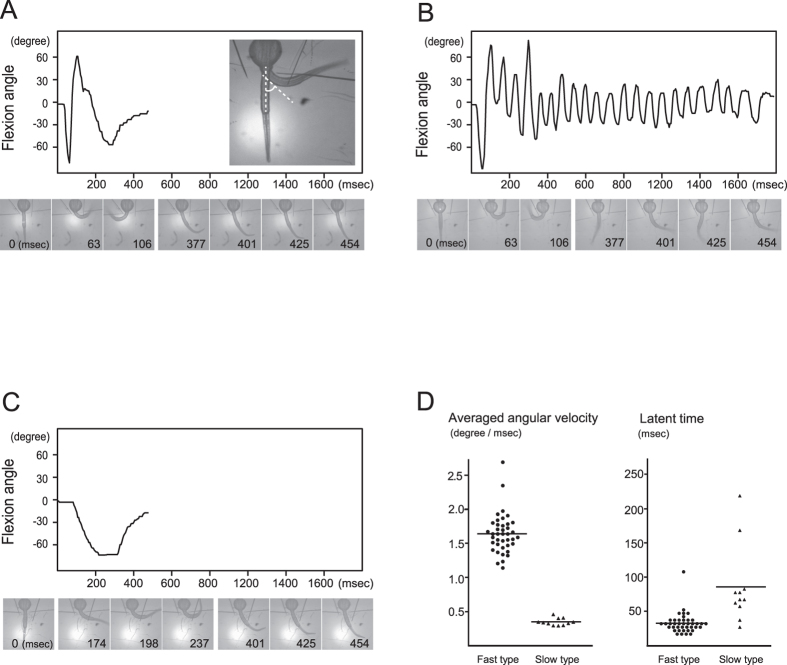
Behaviors elicited by single-cell stimulation of RB neurons. (**A**–**C**) Diagrams of trunk turn angles along a time course after the photostimulation. In most typical cases, zebrafish larvae elicited a pair of alternating turnings (**A**). The turn angle was measured between the midline and the tangent to the curved trunk surface at the proximal yolk tube level (inset of (**A**)). In several samples, turning response was followed by small alternating flips lasting more than one second, which represents the transition from turning to swimming behavior (**B**). In a small number of cases, the trunk flexion was remarkably slower than the above typical response (**C**). (**D**) Scatter plots of averaged angular velocity and latent time for initiation of turning. We categorized response in (**A**,**B**) as "fast type". Response in (**C**) was defined as “slow type” by its angular velocity below 0.5°/msec (fast type, 1.66 ± 0.30°/msec, n = 40 from 10 larvae; slow type, 0.34 ± 0.05°/msec, n = 11 from 7 larvae). Latent time in the fast type response was relatively constant around 30 msec after the stimulation, while it was varied and longer in the slow type (fast type, 29.71 ± 14.29 msec, n = 40 from 10 larvae; slow type, 80.37 ± 54.58 msec, n = 11 from 7 larvae; *P* < 0.01 by Student’s t-test).

Transgenic zebrafish Tg(SAGFF36B/UAS:ChRWR-EGFP) express an optimized channelrhodpsin variant, ChRWR, in approximately 30% of RB neurons in a random and mosaic manner^17^,^18^. We conducted high-speed video imaging of the turning response of 1.5-day-old larvae, elicited by point laser irradiation (φ 0.3 μm) on a single RB cell body. We traced the turning angle between the straight midline and the tangent to the curved line of the trunk (Fig. 1A, inset) for 104 neurons from 10 individual larvae. In most cases (87/104), the response was an immediate C-shaped body turn followed by one or two alternating flips, resembling behavior elicited by physical epidermal contact (Fig. 1A). The alternating flips occasionally continued for more than one second (Fig. 1B; 3/104 cases), indicating a transition from C-shaped turning to brief swimming. In a few other cases, the photostimulation caused slow and coarse body flexion without subsequent flips (Fig. 1C; 14/104 cases). Based on the apparent decrease in angular velocity and prolonged behavioral onset after the stimulation (Fig. 1D), the slow and coarse flexion seems to be dissociated from typical behavior observed in this developmental stage, and might reflect degeneration of the RB neurons involved^19^,^20^ or insufficient synapse formation^21^. In this study, we evaluated the most common behavior (fast type; 90/104 cases) as the typical turning response, and withheld further analysis for the slow and coarse response.

To our surprise, turning began from the ipsilateral side of stimulated neurons in 74 of the 90 typical responders, opposing the touch-response that begins with contralateral turning^6^. When the same transgenic larvae were examined for the physical-touch response by tungsten needle, the turning was 100% contralateral (n = 8 larvae). These results together indicate that ipsilateral turning is a consequence of a single-cell photostimulation that is distinct from the touch response reported in previous studies^6^,^8^,^16^.

### Variable turning strength in the A-P position of RB neurons

**Figure 2:**
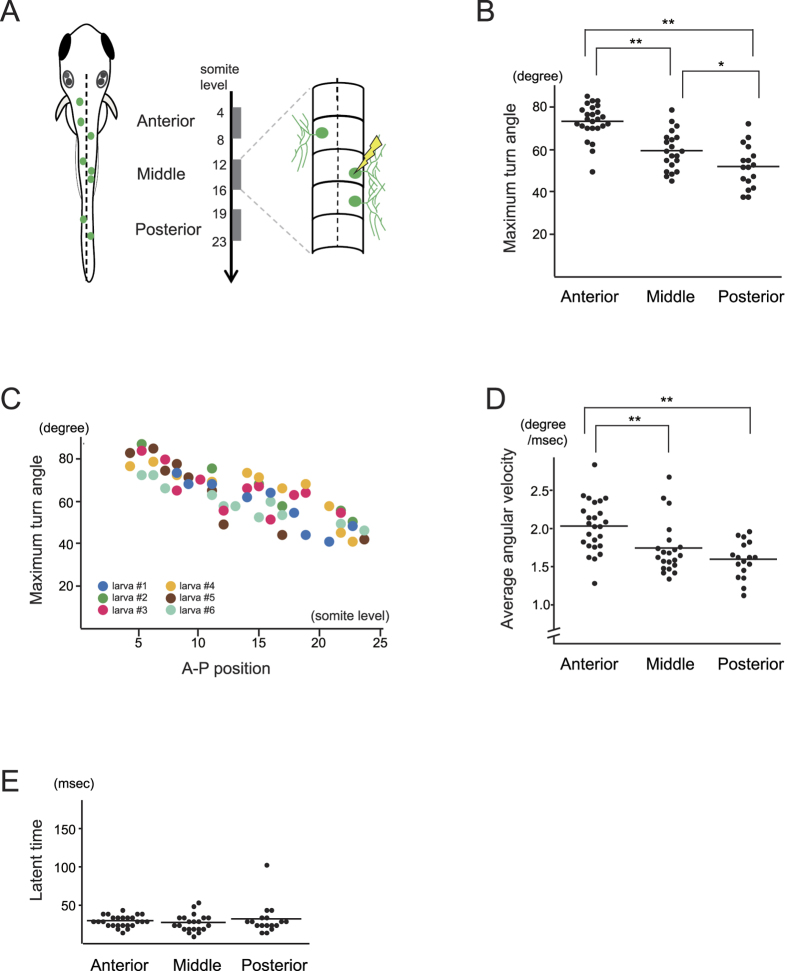
Variable strength of the first turning in single-cell stimulation depending on the A-P position of RB neurons. (**A**) Optimized chanelrhodopsin ChRWR-GFP is expressed by RB neurons in a mosaic manner (green circles). RB neurons were categorized into three groups by their A-P position; Anterior (4–8 somite level, n = 25 neurons from 10 larvae), Middle (12–16 somite, n = 21 from 10 larvae), and Posterior (19–23 somite, n = 17 from 10 larvae), and were subjected to photostimulation. (**B**) Analysis of variance in maximum turn angle elicited by single-cell stimulation. More anterior RB neurons elicited larger turning than middle and posterior neurons (Anterior, 73.0 ± 7.55°; Middle, 60.19 ± 8.48°; Posterior, 53.24 ± 9.26°). Differences in mean values were assessed by the Bonferroni multiple comparison test. *and **denote *P* < 0.05 and *P* < 0.01, respectively. (**C**) Maximum turn angles are plotted along A-P position of stimulated neurons from six individual larvae in different colors. The change in the degree was graded depending on the position. (**D**) Averaged angular velocity was larger in anterior neurons (Anterior, 2.07 ± 0.35°/msec; Middle, 1.77 ± 0.35°/msec; Posterior, 1.62 ± 0.25°/msec). Statistical values as in B. E: Latent time did not show significant difference by the A-P position (Anterior, 29.57 ± 7.15 msec; Middle, 27.15 ± 10.96 msec, Posterior, 31.83 ± 19.7 msec).

**Figure 3:**
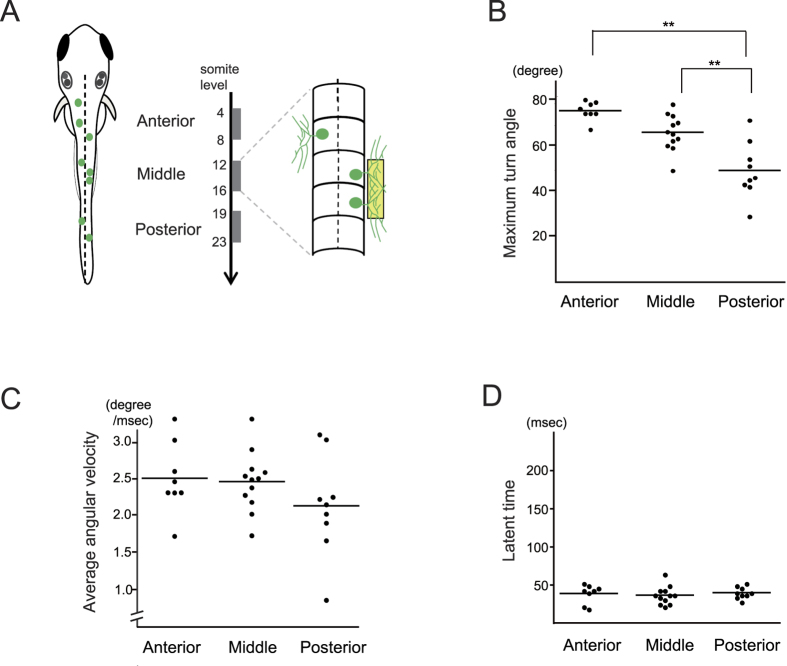
Variable strength of the first turning in multi-cell stimulation along the A-P position of RB neurons. (**A**) Overlapping peripheral arbors from RB neurons were subjected to photostimulation. In a 96 × 12 μm rectangle from the dorsal view, axons from two or three ChRWR-GFP neurons were included, and the stimulated area was categorized into three groups by their A-P position; Anterior (4–8 somite level, n = 8 larvae), Middle (12–16 somite, n = 12 larvae), and Posterior (19–23 somite, n = 9 larvae). (**B**) Analysis of variance in maximum turn angle with multi-cell stimulation. The anterior and middle neurons elicited larger turning angles than the posterior neurons (Anterior, 77.25 ± 4.1°; Middle, 67.67 ± 7.87°; Posterior; 51.22 ± 12.2°). **P < 0.01 by the Bonferroni multiple comparison test. (**C**,**D**) Differences in averaged angular velocity and latent time were not significant in multi-cell stimulation along the A-P position by the Bonferroni test.

**Figure 4:**
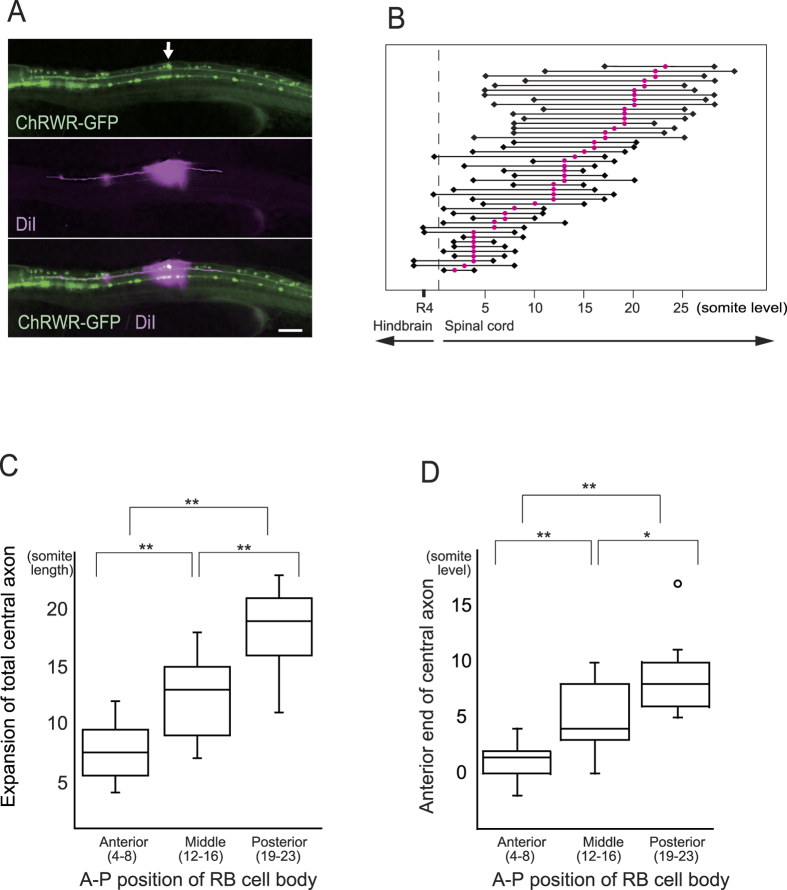
Extent of RB central axons along different A-P cellular position. (**A**) (Upper panel) Ionophoretic diI injection was guided with GFP expressed by RB neurons (arrow). Scale bar 100 μm. (Middle) Ascending and descending axons were fluorescently labeled by anterograde fluid expansion. (Lower) Merged image of GFP and diI fluorescence. (**B**) Extent of RB central axon extension from each individual neuron. Red circles denote cell bodies, and black rhombuses indicate ends of ascending and descending axons. Only small numbers of axons reached the hindbrain level, with the axon endings showing variation even among neurons at the same A-P level, whereas anterior neurons tended to extend more anteriorly. (**C**) Sum length of ascending and descending axons in three A-P groups of RB neurons. Posterior neurons extend longer axons within the spinal cord. Box plots show, 25, 50, and 75th percentiles (boxes) and 2.5 and 97.5th (whiskers). Mean values were 7.5, 13.0, and 19.0 for anterior (n = 12), middle (n = 13), and posterior neurons (n = 13), respectively. **P < 0.01 by Bonferroni’s multiple comparison test. (**D**) Comparison of anterior ends of central axons. Topographic A-P arrangement was statistically maintained within the spinal cord. Mean values were 1.5, 4.0, and 8.0 from the anterior, and numbers of neurons were as in C. *and **denote *P* < 0.05 and *P* < 0.01 by Bonferroni’s multiple comparison test. Open circle indicates outlier.

Whereas the single-cell photostimulation elicited a unique feature in turning direction, the first turning angle varied depending on the A-P position of the neurons. When RB neurons were divided into three groups based on A-P level (Fig. 2A), stimulus of the more-anterior neurons caused a larger turn (Fig. 2B; n = 25, 21, and 17 neurons from 10 larvae for the anterior, middle, and posterior group, respectively). The increase in turning angle by anterior neurons also showed a graded behavior among the individual larvae (Fig. 2C; n = 6). We also tested if the same A-P difference occurs under a condition that resembles epidermal sensation using a pattern-illumination device attached to the microscope. When overlapping multicellular peripheral axons on the epidermal region were subjected to targeted illumination, turning direction was contralateral in all cases (n = 29) and a larger turning angle was again observed in more-anterior neurons (Fig. 3B; n = 8, 12, and 9 larvae for the anterior, middle, and posterior level). These results suggest A-P position is perceived by RB neurons to control behavioral strength. It is known that vertebrate sensory systems form topographic axonal projection to send positional information to neural centers for perception^9^, thus we next examined if the central axons of RB neurons form topographic structure that could convey A-P information. Each RB neuron was fluorescently labeled by intracellular diI injection, and the extent of central axon extension along the DLF was scored (Fig. 4A). In the developmental stage we examined behavioral responses, only small numbers of the central axons (6/42 neurons) reached the hindbrain, and posterior neurons showed longer axons than anterior neurons (Fig. 4B,C; n = 12, 13, and 13 neurons for the anterior, middle, and posterior groups, respectively). Meanwhile, anterior RB neurons extended their central axons more anteriorly within the spinal cord, maintaining the correlation between their receptive field and axonal projection (Fig. 4D; n = 12, 13, and 13 neurons as in Fig. 4C). These results indicate that RB neurons do not form direct topographic projections beyond the spinal cord. A-P information from RB neurons therefore seems to be either transferred to other spinal interneurons that send axons to the supraspinal level or processed within the spinal cord to control behavioral strength.

### Different turning direction is produced by a quantity-sensitive pathway in neural circuits

**Figure 5:**
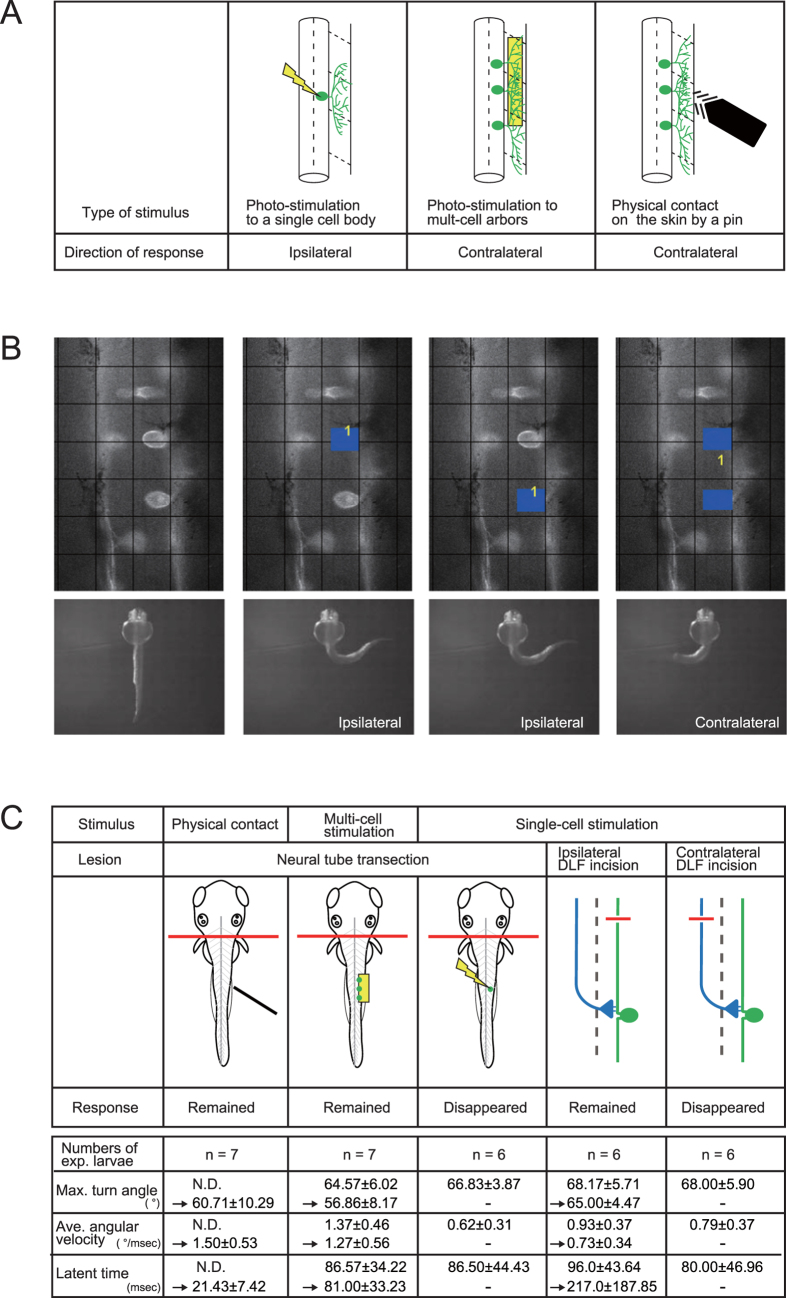
Single- and multi-cell stimulation elicit opposite lateralities by launching different neural circuits. (**A**) Turning direction of the first turning response. Photostimulation of a single RB neuron elicited ipsilateral turning, while photostimulation of peripheral arbors from multi-cell or touch stimulation by pin produced contralateral turning. (**B**) Different laterality is evoked in response to the quantity of stimulated neurons. (Upper panels) RB neurons captured from the dorsal view using a pattern-illumination device. Two neurons on the right side were targeted. Photostimulated areas are marked by blue rectangles in three images on the right. Grid intervals, 24 μm. (Lower panels) Elicited turning responses corresponding to the upper panels. Single-cell photostimulation on its cell body elicited ipsilateral turning, while the same neurons produced the contralateral response by simultaneous stimulation of both cells. Maximum turn angles, averaged angular velocities, and latent times were not significantly different between single- and two-cell stimulations (single cell, n = 12 neurons from 6 larvae, 58.33 ± 5.91°, 0.63 ± 0.52°/msec, 208.75 ± 85.13 msec; two-cell, n = 6 pairs of neurons from 6 larvae, 56.33 ± 6.98°, 0.58 ± 0.29°/msec, 251.00 ± 159.63 msec) (**C**) Summary of lesion experiments for the turning response. Neural tube transection at the second-somite level did not disturb the contralateral response by physical contact or multi-cell neural stimulation (left two columns), but it abolished the ipsilateral response by single-cell stimulation (middle). Incision of the contralateral, but not ipsilateral, DLF abolished the ipsilateral response (right two columns), suggesting that commissural ascending interneurons convey sensory information beyond the spinal cord to the hindbrain. Before and after the lesion operation, quantitation of the parameters of response (attached table) did not show statistical difference by Student’s t-test.

The results presented thus far indicate that turning direction varies in a stimulation mode-dependent manner such as photostimulation of single RB neurons eliciting ipsilateral turning, whereas stimulation of multicellular peripheral arbors or their epidermal contacts produces a contralateral response (Fig. 5A). We therefore hypothesized that turning direction in individual larvae is determined by the numbers of stimulated neurons, and tested pairs of RB neurons for single- and multi-cell stimulation under the pattern illumination device (Fig. 5B). Photostimulation of the cell body of single neurons produced ipsilateral turning in 100% of cases, whereas simultaneous stimulation of both neurons in the pair produced contralateral turning (14 pairs, 28 neurons).

From these results, we speculated that distinct sets of interneurons are activated in response to single- versus multi-cell stimulation. Studies of the zebrafish touch response suggested that reflex circuits are composed of spinal cord neurons, based on the turning response being maintained in lesion experiments following hindbrain removal. On the other hand, hindbrain RS neurons that play central roles in escape behavior of older fish are in place, and spinal cord neurons such as RB and CoPA extend their ascending axons to the hindbrain at the stage we examined the present study^10^,^11^,^22^. To test the possibility that hindbrain neurons are involved in the ipsilateral response by a single RB neuron stimulation, we evaluated susceptibility to lesioning of the hindbrain. The contralateral response elicited by multi-cell stimulation or physical contact was maintained after transection at the second somite level as reported in previous lesion studies (Fig. 5C, left two columns; n = 7 larvae)^8^,^16^. However, single-cell stimulation produced no behavioral response after the transection, indicating that supraspinal neural networks are necessary for the ipsilateral turning (Fig. 5C, middle column; n = 6 larvae). In addition, two candidate pathways seem to be involved in the transmission of RB neuronal activity to the hindbrain beyond the spinal cord (Fig. 5C, right two columns); one is direct projection by the RB central axons that run along the ipsilateral DLF, and the other is indirect via synaptic connection to CoPA interneurons whose post-commissural axons run anteriorly along the contralateral DLF^8^. To clarify which side of the DLF transmits such RB sensory information, we made an incision on either the GFP-labeled ipsilateral DLF or the contralateral pathway. Although the former incision (ipsilateral DLF disconnection) robustly reproduced the single cell-evoked response, the latter (contralateral disconnection) abolished the characteristic turning response (n = 6 larvae for each side of the tract). The data were also consistent with our RB axon tracing analysis (Fig. 4B), in which only small numbers of the RB anterior axons extended beyond the spinal cord, suggesting an interneuronal signal relay to the supraspinal circuits. Thus our results strongly implicate the contralateral DLF as a transmission pathway for sensory input from RB neurons to the hindbrain beyond the spinal cord, and suggest that supraspinal neural components are involved in the ipsilateral turning response triggered by single-cell RB neuronal stimulation.

## Discussion

Studies of adult fish behavior generally described a tight correlation between the stimulus direction and initial body turning strength. Water vibration from the front evokes a larger turn than that from the back, as suited for avoidance trajectories in response to a threat^23^. Our results now show that RB neurons adopt a similar behavioral principle, driving a larger turning angle in response to anterior stimulus, occurring from the first appearance of escape behavior in developing zebrafish larvae. Where and how A-P difference is transferred for behavioral strength remains an intriguing question. In vertebrate somatosensory systems, topographic axonal projections to higher centers reportedly perceive positional information^9^. However, our analysis of RB central axons indicates that most ascending branches terminate within the spinal cord. Palanca *et al.*^24^ also reported that posterior RB neurons do not send central axons beyond the spinal cord, suggesting that direct topographic projection is not involved. Rather, it is more likely that other spinal interneurons relay sensory A-P information to higher centers, and accumulating evidence is emerging to implicate CoPA neurons as the sensory interneurons responsible for trunk mechanical sensation. Indeed, CoPA neurons that reside in the dorsolateral spinal cord and send commissural ascending axons along the contralateral DLF were reported to form synaptic connections with RB central axons^8^,^10^,^12^,^13^, while electrophysiological studies further indicated that CoPA neurons mediate sensory input, with glutamate-driven action potential and glycinergic corollary discharge recorded after touch-stimulus^8^,^25^. The present lesion studies also support that the contralateral ascending tract, which includes CoPA axons, is responsible for transmitting RB activity to supraspinal circuits (Fig. 5C), and that A-P information is probably relayed by this type of interneuron. Alternatively, the A-P difference is transferred for behavioral strength within intraspinal circuits, and our present results showed RB ascending axons maintaining the A-P correlation within the spinal cord and a longer total axon length in posterior RB neurons (Fig. 4C,D). These anatomical features might therefore play a part in regulating behavioral strength through as yet unknown intraspinal circuits. At the premotor level that would generate difference in the trunk muscle contraction, RS neurons in the hindbrain and CiD (circumferential descending) neurons in the spinal cord were reported to exhibit different firing patterns between head and tail touch stimulation, implicating their involvement in behavioral strength regulation^15^,^26^. If and how A-P information from RB neurons could affect the activities of these neurons is an interesting issue, and should be examined in future studies.

**Figure 6:**
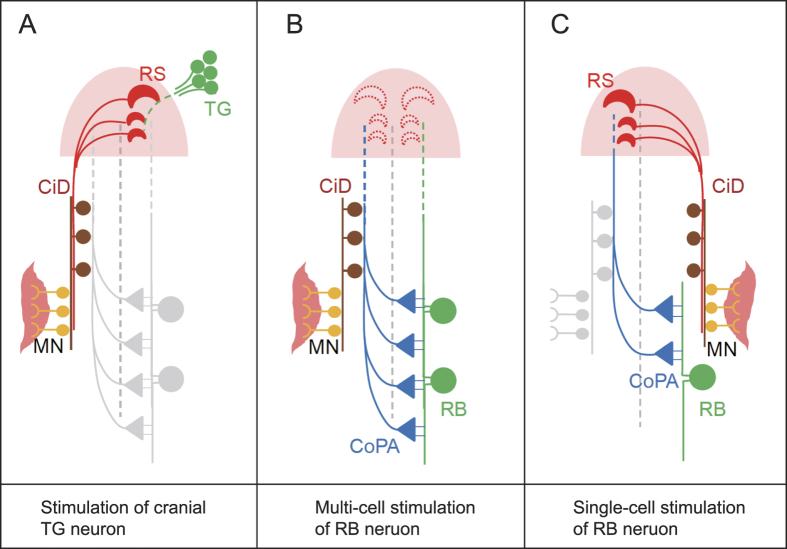
Diagram of neural circuits that produce contra- and ipsilateral turning depending on the input quantity. (**A**) Turning behavior elicited by head touch stimulus. Trigeminal sensory neurons (TG) activate hindbrain RS neurons at the stimulated side, and commissural descending axons from RS neurons excite contralateral motor neurons (MN) directly and indirectly through spinal interneurons. (**B**) Contralateral turning by trunk touch and RB multi-cell stimulation is triggered by an intraspinal reflex circuit. RB neurons form synapses with CoPA neurons, and their commissural axons send sensory signal to the opposite side of the spinal cord. Descending interneurons such as CiD were shown to excite motor neurons for escape; however, it remains unclear how these pathways are connected, although contact between CoPA axons and CiD neuron were suggested^8^. (**C**) The ipsilateral response requires supraspinal neural circuits. Single-cell RB input activates CoPA neurons as in B, but is proposed to be fewer than with the multi-cell input. If small numbers of CoPA neurons do not launch the intraspinal reflex, yet transmit the signal beyond the spinal cord through the contralateral DLF, hindbrain RS neurons might trigger turning behavior by driving motor neurons on the stimulated side.

In this study, we showed that larval turning direction is switched in response to varied numbers of stimulated neurons. Multi-cell stimulation to RB neurons elicited turning to the contralateral side as seen in the touch-evoked response, and single-cell stimulation drove a novel type of turning ipsilateral to the stimulus. Epidermal sensory innervation is divided into trigeminal neurons on the head and RB neurons on the trunk, whereas the neural pathway from RB neurons is less understood. In the turning response evoked by the cranial sensory pathway, hindbrain Mauthner and other RS neurons are activated on the stimulated side, and their commissural descending projections excite spinal motor neurons directly and indirectly through descending interneurons^15^,^26^,^27^,^28^,^29^ (Fig. 6A). Hindbrain RS neurons are also activated by trunk touch stimulation, even with different extents of head stimulation^15^, suggesting the sensory input is transferred to these neurons. However, lesion studies of hindbrain removal showed preservation of the trunk touch response in spinalized larvae^8^,^16^ (Fig. 5C), indicating that a reflex circuit composed of spinal cord neurons should be able to produce the turning response. Among approximately ten spinal neurons identified at this developmental stage, CoPA neurons receive synaptic transmission from RB neurons as mentioned above. Other studies also reported the activity of several descending interneurons such as CiD and IC (ipsilateral projecting) neurons during touch-evoked response as well as during spontaneous swimming activity^26^,^30^,^31^. These descending neurons form electrical connections by gap junction to synchronize neural activity between them and between CiD and motor neurons, and also form glutamatergic synapses that excite motor neurons^29^,^32^. Potential connections of the contralateral CoPA axons with CiD and IC cells were suggested to link these ascending and descending components^8^, although it remains to be elucidated how these descending neurons are activated by CoPA (Fig. 6B).

In contrast to the contralateral turning elicited by multi-cell stimulation of RB neurons, the ipsilateral response with single-cell stimulation exhibited different lesion susceptibility, indicating that a distinct circuitry involving supraspinal neurons executes the turning behavior. While the present study was limited in specifying all the neural components involved, the requirement of the opposite side of the DLF (Fig. 5C) implicates this pathway in transmitting the turning signal, probably together with CoPA axons connecting to supraspinal circuits. Although CoPA neurons are also involved in the intraspinal circuit, the single-cell stimulation might activate fewer numbers of CoPA neurons than the multi-cell stimulus. Thus, if a single-cell signal is insufficient for launching the intraspinal circuits, but is further transmitted beyond the spinal cord through the opposite side of the DLF, the ascending signal might then activate nearby RS neurons in the opposite side, providing excitation of motor neurons on the stimulated side (Fig. 6C). Further morphological and electrophysiological investigation is necessary to elucidate neural circuits driving the ipsilateral turning, although the present study suggested a model that includes a neural switch between intra- and supraspinal circuits depending on input quantity from the sensory neurons.

Fish escape behavior is an immediate and robust startle reflex to significant threat, while harmless minor sensation or irrelevant noise should be filtered. In this regard, the requirement of multi-cell input for the robust intraspinal reflex makes sense in achieving escape by contralateral turning. What the ipsilateral response represents in larval zebrafish life remains an open question. For example, ipsilateral turning may help in capturing small planktonic animals that make minimum water vibration, or it may indicate cross talk with neural information for the A-P recognition, which might be suppressed in matured neural circuits. Although the current study is limited to the examination of early larval stages only, with transgenic ChRWR expression downregulated after those stages, it would be interesting to see if the ipsilateral response remains in later larval stages, juvenile, and adult zebrafish.

## Conclusion

Zebrafish RB neurons perceive mechanical sensation, and A-P positional information on the trunk region is processed for coordinating behavioral strength to take appropriate escape direction. Multi-cell stimulation on RB neuron is required for robust contralateral turning that is triggered by intraspinal reflex. On the other hand, less stimulation by single RB neuron elicits a novel type of ipsilateral turning, although the behavioral significance of this observation remains to be elucidated. Our results indicate that sensory input from RB neurons is processed differently for A-P recognition and triggering different types of behavior, through distinct neural circuits.

## Methods

### Zebrafish colony

Zebrafish were maintained in the laboratory fish room under a 14/10-h light/dark cycle. The larvae were maintained at 28.5 °C, with the developmental stages determined as previously described^33^, and expressed in hours post-fertilization (hpf) or days post-fertilization (dpf). We previously established the Tg(SAGFF36B; UAS:ChRWR-EGFP) double transgenic line used in this study, using a GAL4 driver strain Tg(SAGFF36B) provided by the NBRP (National BioResource Project, Japan)^18^. Larvae were treated with 0.2 mM phenylthiourea (Sigma-Aldrich, St. Louis, MO) at approximately 24 hpf to inhibit pigmentation and were used for the behavioral experiments. The use of these animals for experimental purposes was conducted according to the guidelines for the care and use of laboratory animals of Tohoku University, and approved by the committee of laboratory animal experiment of Tohoku University.

### Behavioral analysis

The zebrafish larval turning response was examined at 30–34 hpf when spontaneous twitching had ceased. The larvae heads were then embedded in 2.0% LMP (low melting point) agarose (Nippon Gene Co., Ltd.), and their trunks and tails were left to move freely in 1/3 diluted Ringer’s solution (38.7 mM NaCl, 0.97 mM KCl, 0.6 mM CaCl_2_, 1.67 mM HEPES, pH 7.2). Laser irradiation (Figs 1 and 2) was performed under upright confocal laser microscopy (A1R, Nikon corp.), which focused on ChRWR-GFP-expressing cell bodies with a 0.3 μm diameter beam of 405 nm wavelength. To reduce photobleaching artifact and cytotoxic damage from the irradiation, the first response in each neuron was evaluated by raising laser power in steps at 0.28, 0.53, 0.74, 1.12, 1.67, and 2.6 mW for 20 msec. A pattern-illumination device (L-StimHGLGP-XL4 with BX51WI, Olympus corp.) was used for stimulating peripheral axons from multiple neurons (Fig. 3) and for cell bodies to examine single- and multi-cell induced responses (Fig. 5). 460–495 nm wavelength light from a high-pressure mercury lamp was controlled onto a rectangle of 96 × 12 μm (peripheral axon area) or 15 × 18 μm (cell body) using the device at 23.3 mW/mm^2^. To minimize light exposure, the first response was evaluated by increasing the irradiation time in steps at 50, 100, and 200 msec. Behavioral images were captured through a pathway attached under the microscope stage and recorded with high-speed cameras at 207 fps (ICL-B0620M-KC, ARGO Corp.) or 333 fps (EoSens MC1362, Mikrotron GmbH). These sequential images were analyzed with Matlab (The MathWorks, Inc.) to measure maximum turning angle of the larvae between the midline and the tangent to the curved trunk surface at the proximal yolk tube level (Fig. 1A inset). Averaged angular velocity was determined from a whole flexion episode (from the onset of flexion to the maximum turn). In Fig. 1D, the “slow type” response was observed in relatively caudal neurons, thus only values from 15- to 25-somite level neurons were plotted. In Fig. 5B, targeted neurons were selected from 10–18 somite level. Statistical differences were analyzed using Student’s t test for two groups and ANOVA with Bonferroni correction for post hoc testing.

### Lesion study

Larvae were anesthetized in 0.02% 3-aminobenzoic acid ethylester (Sigma-Aldrich Co. LLC.) and embedded in 2% LMP agarose. The neural tube was incised at the second-somite level using a razor blade under stereomicroscopy, and its complete disjunction was confirmed at the end of experiment following fixation in 4% paraformaldehyde (PFA; Nakalai Tesque, Inc.). Transection of the DLF was performed under an upright microscopy (Axio Examiner D1, Carl Zeiss GmbH) using a sharp glass capillary attached to a micromanipulator (Leica Microsystems GmbH). The DLF was labeled with GFP to guide the accurate incision and minimize artificial destruction of surrounding tissues.

### Anterograde labeling of RB central axons

Anesthetized larvae were embedded in 1% agar, soaked in ice-cold 4% PFA in PO_4_ buffer (0.1 M, pH 7.3) for 2 minutes, and then washed three times with PO_4_ buffer. GFP-positive RB cell bodies were punctured with a fine electrode and 10 mg/ml DiIC18(3) (Molecular Probes, Thermo Fisher Scientific Inc.) in N,N-dimethylformamide (ICN Pharmaceuticals, Inc.) was iontophoretically injected. Larvae were fixed overnight at room temperature with 4% PFA, and then the extent of each labeled axon was measured using an all-in one fluorescence microscope (BZ9000, Keyence Corp.).

## Additional Information

**How to cite this article**: Umeda, K. *et al.* Position- and quantity-dependent responses in zebrafish turning behavior. *Sci. Rep.* **6**, 27888; doi: 10.1038/srep27888 (2016).
